# Supplementary material for: Tailoring Epoxy Network Architecture and Stiffness-Toughness Balance Using Competitive Short- and Long-Chain Curing Agents: A Multiscale Simulation Study
Source: Polymers (Basel). 2025 May 9;17(10):1297. doi: 10.3390/polym17101297 (PMC12114751; doi:10.3390/polym17101297)
Supplement: Supplementary file 1 [file polymers-17-01297-s001.zip › polymers-3599969-supplementary.pdf]

Tailoring Epoxy Network Architecture and Stiffness-Toughness Balance Using Competitive Short- and Long-Chain Curing Agents: A Multiscale Simulation Study

Zhiyong Dong [1,2], Yuqing Li [1], Renhai Huang [1], Xuze Zhang [1], Mingyang Li [1], Duo Liu [1],

Rui Shi [1], Xuanbo Zhu [2,*], Jianxin Mu [2] and Hujun Qian [1,2,*]

[1] State Key Laboratory of Supramolecular Structure and Materials, Institute of Theoretical Chemistry, College of Chemistry, Jilin University, Changchun 130012, China; dongzy21@mails.jlu.edu.cn (Z.D.); yuqingl23@mails.jlu.edu.cn (Y.L.); huangrh24@mails.jlu.edu.cn (R.H.); zhangxz20@mails.jlu.edu.cn (X.Z.); lmy23@mails.jlu.edu.cn (M.L.); liuduo24@mails.jlu.edu.cn (D.L.); shirui816@jlu.edu.cn (R.S.)

[2] Key Laboratory of High Performance Plastics, National & Local Joint Engineering Laboratory for Synthesis Technology of High Performance Polymer, College of Chemistry, Jilin University, Ministry of Education, Changchun 130012, China; jianxin_mu@jlu.edu.cn

***** Correspondence: zhuxuanbo@jlu.edu.cn (X.Z.); hjqian@jlu.edu.cn (H.Q.)

Table of Contents

THEORETICAL CALCULATION DETAILS 2
DFT Calculation. 2
Reaction Rate Constant. 3
Atomistic Simulation. 4
DEVELOPMENT OF COARSE-GRAINED (CG) POTENTIALS 5
Potentials of Non-bonded Interaction. 5
Potentials of Bonds. 6
CG SIMULATION OF THE EPOXY RESIN CURING PROCESS. 10
CG Reaction Procedure. 10
References 12

THEORETICAL CALCULATION DETAILS

DFT Calculation. Electronic structure calculations were performed using Gaussian09[1] software package. Geometric optimizations and frequency calculations for all reactants, intermediates, transition states, and products were performed at the B3LYP[2]/6-311G(d)[3]level. Intrinsic reaction coordinate (IRC)[4] calculations confirmed each transition state had a single imaginary frequency. Single-point energy calculations were performed with the M06-2X[5] functional functional and the def2-TZVP[6] basis set on geometries optimized at the B3LYP/6-311G(d) level. The reported energy is the Gibbs free energy referenced to the reactants, combining electronic energy from single-point calculations and thermodynamic corrections.

Reaction Rate Constant. The rate constant k is calculated according to Transition State Theory (TST)[7-9]:

$$k = \sigma \frac{k_B T}{h} \left(\frac{RT}{P_0}\right)^{\Delta n} \exp\left(-\frac{\Delta G^{\neq}}{k_B T}\right) \qquad (1)$$

Here, σ represents the degeneracy of the reaction path, with a value of 1 in this system; k_B is the Boltzmann constant; T is the reaction temperature; h is the Planck constant; R is the gas constant; P_0 is the pressure; Δn for a bimolecular reaction is 1, and $\Delta G^{\neq}$ is the activation Gibbs free energy.

Atomistic Simulation. In this study, the reference system was simulated using the GROMACS[10-12] software under the OPLS-AA[13, 14] force field. The system comprises 100 DABPB and 100 mPDA molecules, forming 200 4-1 epoxy oligomers, confined within a cubic box and subject to three-dimensional periodic boundary conditions. The systems underwent initial energy minimization employing the steepest descent method until reaching convergence with a maximum force of 100 kJ/(mol.nm). All non-bonded interactions with an intra-chain separation of no more than three bonds were excluded. Constraints on bond lengths were implemented using the LINCS algorithm[15]. The Lennard-Jones potential[16] was employed, and the electrostatic interactions were addressed using the particle-mesh Ewald (PME) algorithm[17, 18], employing a cutoff of 1.2 nm for both van der Waals (VDW) and short-range electrostatic interactions. The simulation time step was set to 1 fs. Temperature and pressure were controlled using the v-rescale thermostat[19] and Parrinello-Rahman barostat[20], respectively. In the initial stage, NPT ensemble simulations were conducted at 493 K and 1 atmosphere for 200 ns to ensure density and structural convergence of the system. Subsequently, NVT ensemble simulations were performed for 40 ns to achieve configuration equilibrium, and the last 20 ns of the trajectory were used for statistical analysis of the properties of the reference system.

DEVELOPMENT OF COARSE-GRAINED (CG) POTENTIALS

Potentials of Non-bonded Interaction. Intermolecular interactions were described by a Lennard-Jones (12-6) potential, calibrated to reproduce the density at room temperature.

Table S1. Simulation parameters of $\boldsymbol{\varepsilon}$ used in our simulation.

σ	A	B	X	Y	Z
A	0.6	0.5	0.5	0.5	0.45
B		0.4	0.4	0.4	0.35
X			0.4	0.4	0.35
Y				0.4	0.35
Z					0.3

Parameters used in the simulation, where ε =1.8 represents the depth of the potential well and r_c = 2.0 denotes the cutoff radius for the interaction potential.

Table S2. The density at room temperature of the final cured product from epoxy resin and hardener compositions with varying ratios.

Molar ratio of hardener compositions (DABPB/mPDA)	1/0	3/1	1/1	1/3	0/1
Density (g/cm^3)	1.212	1.235	1.253	1.280	1.301

The cured network density of DABPB/mPDA=1/0 (1.212 g/cm^3) is marginally lower than that of the reference epoxy system DGEBA/DDS (1.237 g/cm^3)[21], which can be primarily ascribed to the bulkier molecular structure and flexible ether linkages in DABPB. Conversely, the higher density observed for DABPB/mPDA=0/1 (1.301 g/cm^3) stems from the more compact molecular architecture of mPDA and its greater aromatic content, resulting in denser network packing. These results are fully consistent with theoretical predictions, validating the accuracy of the simulation data.

Potentials of Bonds. In our work, the CG model is constructed based on an AA force field. Its potential energy includes terms for bonds, and angles. The methodology is as follows:

(I) All-atom simulations were performed in the NPT ensemble for 200 ns, followed by 40 ns in the NVT ensemble to achieve equilibrium configurations.

(II) The center of mass (COM) distributions for bond lengths ($p(r)$ and angles (p(θ)) were obtained from the last 20 ns of the NVT trajectory using Gaussian distribution functions (Formula (2)).

This approach captured the equilibrium configurations and key molecular parameters required for the coarse-grained model development.

$$p(r) = y_0 + Ae^{-2\left(\frac{(x-x_0)^2}{w^2}\right)} \qquad (2)$$

$$w = \frac{h/\sqrt{\ln 4}}{2} \qquad (3)$$

In this context, y_0 represents a negligible parameter that exerts no conclusive impact on the distribution functions and can thus be safely disregarded. On the other hand, x_0 denotes the target bond length or angle value, and h corresponds to the width at half-maximum.

(III) The bond potential $\mathrm{U(r)}$, and angle potential $\mathrm{U(\theta)}$ in the CG model were determined using the Iterative Boltzmann Inversion (IBI) method.

$$U_{CG}^{r} = -k_B T \ln \frac{p(r)}{r^2} \qquad (4)$$

$$U_{CG}^{\theta} = -k_B T \ln \frac{p(\theta)}{\sin\theta} \qquad (5)$$

Here, k_B represents the Boltzmann constant, $p(r)$ is the bond length distribution function in the AA system, and $p(\theta)$ is the angle distribution function in

the AA system.

The IBI method was employed. The potential energy was iteratively updated using Equation (6), where the p_i was utilized to modify the potential energy $U^{i+1}(r)$.

$$U^{i+1}(r) = U^{i}(r) + k_B T \ln\left(\frac{p_i}{p_{tar}}\right) \qquad (6)$$

Here, U^{i+1} represents the potential energy at the $(i+1)$-th iteration. The initial potential energy is denoted as $U^{0}(r)$.

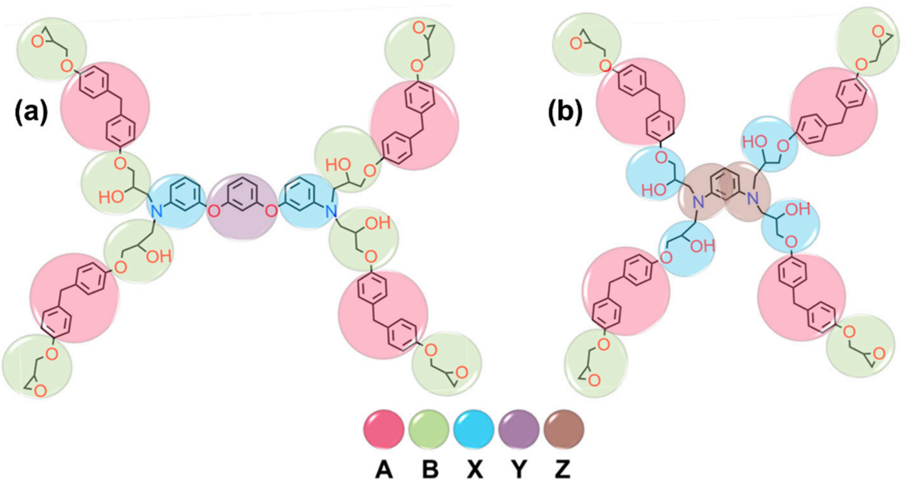

Scheme S1. 4-1 epoxy oligomer (comprising four DGEBF molecules linked via curing agents): (a) with DABPB as the curing agent, (b) with mPDA as the curing agent.

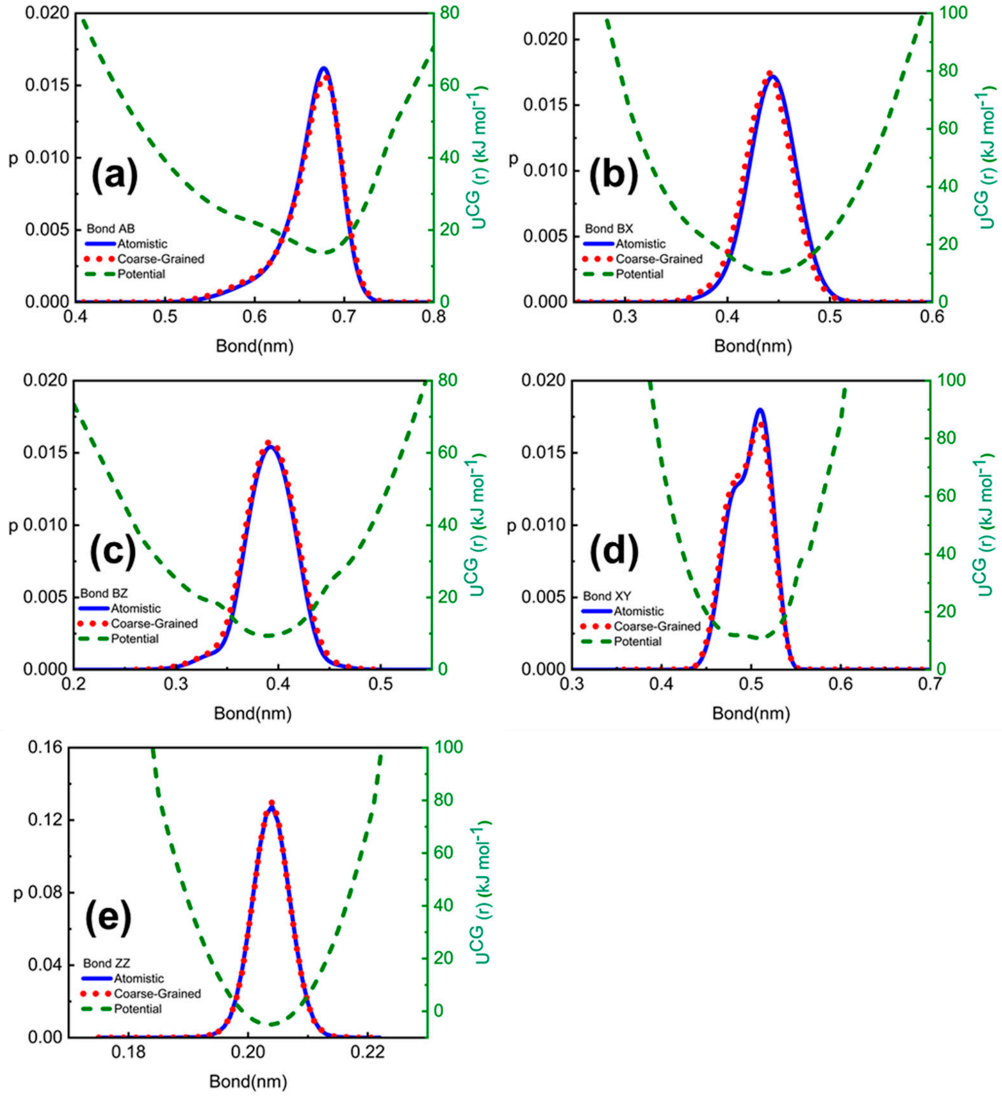

Figure S1. Distributions and potentials of bond lengths for 4-1 epoxy oligomer in CG and AA models.

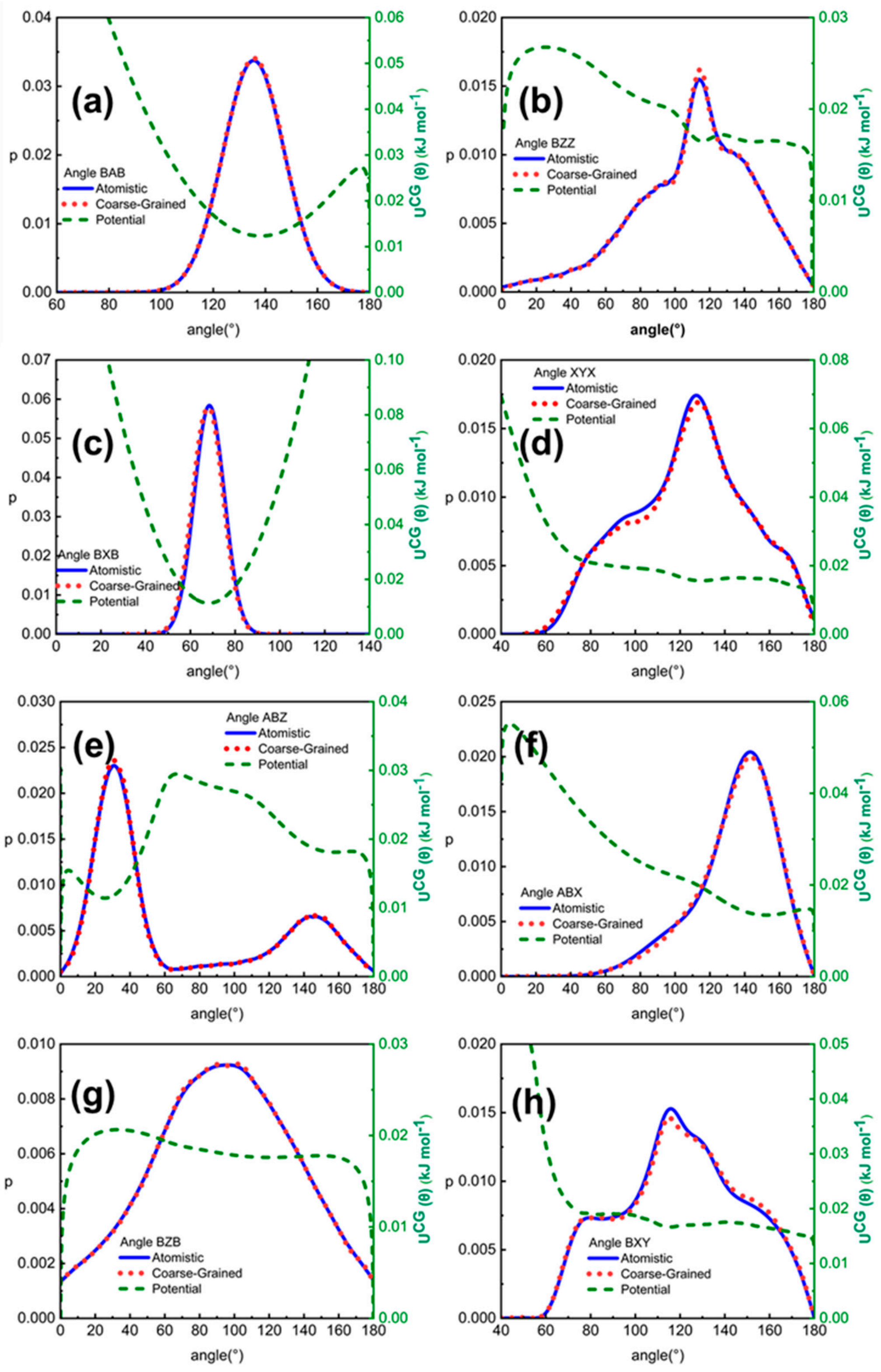

Figure S2. Distributions and potentials of angles for 4-1 epoxy oligomer in CG and AA models.

Based on the above results, our CG model aligns well with the AA system, demonstrating strong consistency.

CG SIMULATION OF THE EPOXY RESIN CURING PROCESS.

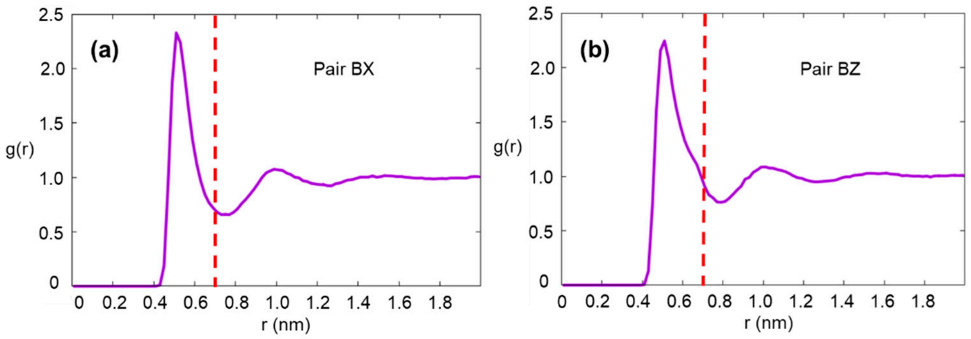

Figure S3. The non-bonded distribution function curves for particles (a) B and X; (b) B and X. The dash lines indicate the rage (<0.7 nm) of the polymerization reactions around a reactive center.

CG Reaction Procedure. We developed a CG reaction model combining molecular dynamics and reactive Monte Carlo methods to study the epoxy resin curing process. Molecular diffusion is modeled by molecular dynamics, while bond formation is handled by reactive Monte Carlo. The reaction is controlled by the capturing radius (r_c), set between 0-0.7 nm based on the first peak of the non-bonded distribution function for particles BX and BZ (Figure S3). Within this range, the nearest complementary particle is selected for reaction, assuming local equilibrium for each molecular conformation. The reaction is governed by the probability parameter Pr. If two particles do not belong to the same unit, a random number $\gamma \in [0,1]$ is generated to determine whether the reaction occurs. When $\gamma < P_r$, the reaction takes place, and the interaction parameters between particles transition from a non-bonded to a bonded state, accompanied by a change in the particle type. Thus, a particle only binds with its opposite type. For a coarse-grained particle X or Z that has not participated in any

reactions, its remaining reaction count is 2. If X or Z has participated in one reaction, such as being an end particle in a polymer chain, its remaining reaction count is 1. If X or Z has participated in two reactions, like being an internal particle in a polymer chain, its remaining reaction count is 0. For a coarse-grained particle B that has not participated in any reactions, its remaining reaction count is 1. If B has participated in one reaction, as an internal particle in a polymer chain, its remaining reaction count is 0. The parameter P_r is a crucial factor regulating the relative reactivity of functional groups. In the polycondensation reaction between coarse-grained particles B and XZ, P_r is determined by the rate constants k of various reaction steps in the epoxy resin curing process, which will be detailed in the Results and Discussion section.

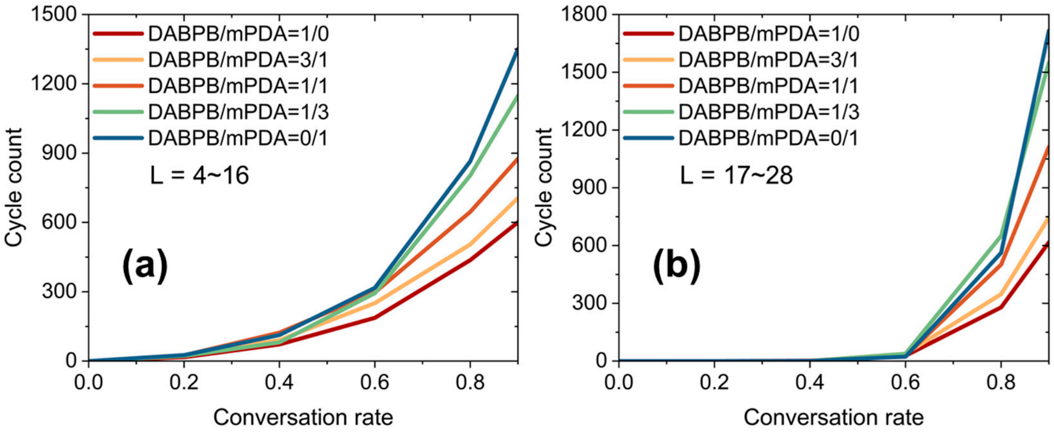

Figure S4. Curves showing the variation in the number of cycles corresponding to different cycle sizes in the network of systems with varying DABPB/mPDA ratios, as a function of conversion rate. (a) Cycle size L = 4~16; (b) Cycle size L = 17~28.

Figure S4 presents the evolution of cycle counts for different cycle sizes in the cured epoxy network as a function of conversion. Figure S4a corresponds to cycle sizes L =

4~16, while Figure S4b covers L = 17~28. The results show that a higher proportion of short curing agent mPDA significantly increases the number of cycles in both size ranges at the same conversion, indicating a higher crosslinking density. Consequently, a higher mPDA content is expected to enhance the modulus of the final cured product.

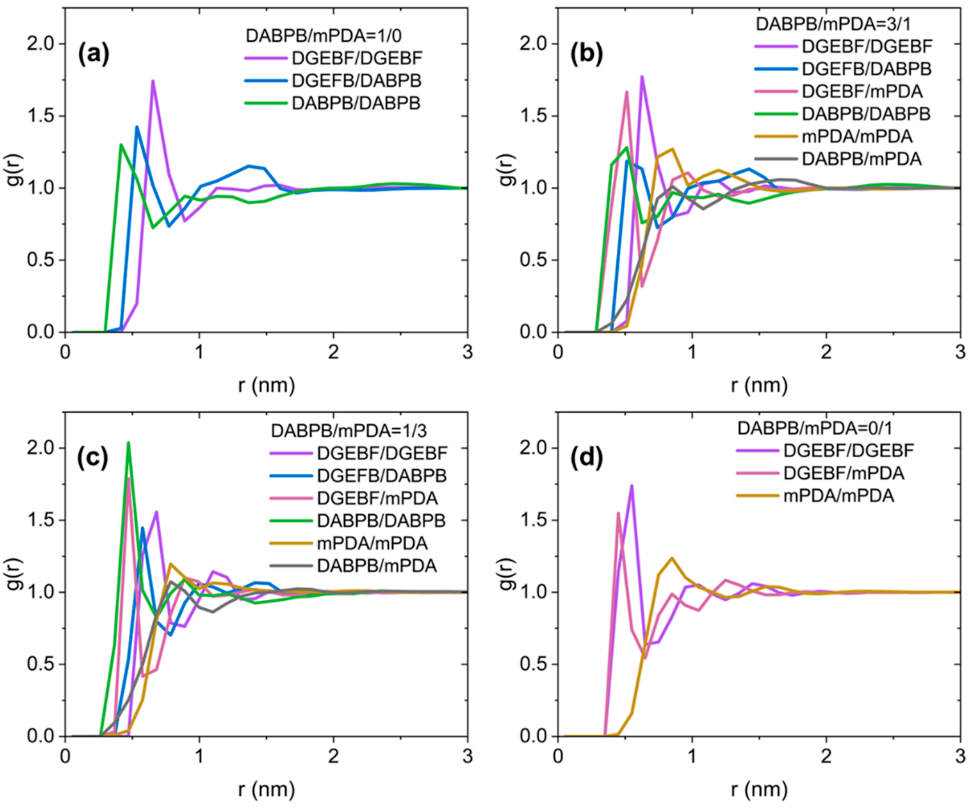

Figure S5. Radial distribution functions between the molecular centroids of individual components in the system with DABPB/mPDA ratio = (a)1/0; (b)3/1; (c)1/3; (d)0/1.

References

(1) Frisch, M. Gaussian 09, Revision D. 01/Gaussian. Inc: 2009.

(2) Becke, A. D. Density-functional thermochemistry. I. The effect of the exchange-

only gradient correction. *The Journal of Chemical Physics* **1992**, *96* (3), 2155-2160.

(3) McCormick, T. M.; Bridges, C. R.; Carrera, E. I.; DiCarmine, P. M.; Gibson, G. L.; Hollinger, J.; Kozycz, L. M.; Seferos, D. S. Conjugated polymers: Evaluating DFT methods for more accurate orbital energy modeling. *Macromolecules* **2013**, *46* (10), 3879-3886.

(4) Fukui, K. Formulation of the reaction coordinate. *The Journal of Physical Chemistry* **1970**, *74* (23), 4161-4163.

(5) Zhao, Y.; Truhlar, D. G. The M06 suite of density functionals for main group thermochemistry, thermochemical kinetics, noncovalent interactions, excited states, and transition elements: two new functionals and systematic testing of four M06-class functionals and 12 other functionals. *Theoretical Chemistry Accounts* **2008**, *120*, 215-241.

(6) Weigend, F.; Ahlrichs, R. Balanced basis sets of split valence, triple zeta valence and quadruple zeta valence quality for H to Rn: Design and assessment of accuracy. *Physical Chemistry Chemical Physics* **2005**, *7* (18), 3297-3305.

(7) Eyring, H. The activated complex in chemical reactions. *The Journal of Chemical Physics* **1935**, *3* (2), 107-115.

(8) Pechukas, P. Transition state theory. *Annual Review of Physical Chemistry* **1981**, *32* (1), 159-177.

(9) Truhlar, D. G.; Garrett, B. C.; Klippenstein, S. J. Current status of transition-state theory. *The Journal of Physical Chemistry* **1996**, *100* (31), 12771-12800.

(10) Hess, B.; Kutzner, C.; Van Der Spoel, D.; Lindahl, E. GROMACS 4: algorithms

for highly efficient, load-balanced, and scalable molecular simulation. *Journal of Chemical Theory and Computation* **2008**, *4* (3), 435-447.

(11) Pronk, S.; Páll, S.; Schulz, R.; Larsson, P.; Bjelkmar, P.; Apostolov, R.; Shirts, M. R.; Smith, J. C.; Kasson, P. M.; Van Der Spoel, D. GROMACS 4.5: a high-throughput and highly parallel open source molecular simulation toolkit. *Bioinformatics* **2013**, *29* (7), 845-854.

(12) Páll, S.; Abraham, M. J.; Kutzner, C.; Hess, B.; Lindahl, E. Tackling exascale software challenges in molecular dynamics simulations with GROMACS. In Solving Software Challenges for Exascale: International Conference on Exascale Applications and Software*, EASC 2014*, Stockholm, Sweden, April 2-3, 2014, Revised Selected Papers *2*, 2015; Springer: pp 3-27.

(13) Jorgensen, W. L.; Tirado-Rives, J. The OPLS [optimized potentials for liquid simulations] potential functions for proteins, energy minimizations for crystals of cyclic peptides and crambin. *Journal of the American Chemical Society* **1988**, *110* (6), 1657-1666.

(14) Jorgensen, W. L.; Maxwell, D. S.; Tirado-Rives, J. Development and testing of the OPLS all-atom force field on conformational energetics and properties of organic liquids. *Journal of the American Chemical Society* **1996**, *118* (45), 11225-11236.

(15) Hess, B.; Bekker, H.; Berendsen, H. J.; Fraaije, J. G. LINCS: A linear constraint solver for molecular simulations. *Journal of Computational Chemistry* **1997**, *18* (12), 1463-1472.

(16) Träskelin, P.; Kuhl, T.; Faller, R. Molecular dynamics simulations of polystyrene

brushes in dry conditions and in toluene solution. *Physical Chemistry Chemical Physics* **2009**, *11* (47), 11324-11332.

(17) Darden, T.; York, D.; Pedersen, L. Particle mesh Ewald: An N· log (N) method for Ewald sums in large systems. *The Journal of Chemical Physics* **1993**, *98* (12), 10089-10092.

(18) Essmann, U.; Perera, L.; Berkowitz, M. L.; Darden, T.; Lee, H.; Pedersen, L. G. A smooth particle mesh Ewald method. *The Journal of Chemical Physics* **1995**, *103* (19), 8577-8593.

(19) Bussi, G.; Donadio, D.; Parrinello, M. Canonical sampling through velocity rescaling. *The Journal of Chemical Physics* **2007**, *126* (1).

(20) Martoňák, R.; Laio, A.; Parrinello, M. Predicting crystal structures: the Parrinello-Rahman method revisited. *Physical Review Letters* **2003**, *90* (7), 075503.

(21) Grillet, A. C.; Galy, J.; Gérard, J.-F.; Pascault, J.-P. Mechanical and viscoelastic properties of epoxy networks cured with aromatic diamines. *Polymer* **1991**, *32* (10), 1885-1891.
